# Optimization of a methodology for speciation of arsenic, selenium and mercury in blood samples based on the deep eutectic solvent

**DOI:** 10.1016/j.mex.2019.09.018

**Published:** 2019-09-18

**Authors:** Reza Akramipour, Mohammad Reza Golpayegani, Mahmoud Ghasemi, Negar Noori, Nazir Fattahi

**Affiliations:** a School of Medicine, Kermanshah University of Medical Sciences, Kermanshah, Iran; b Clinical Research Development Center, Imam Khomeini and Mohammad Kermanshahi and Farabi Hospitals, Kermanshah University of Medical Sciences, Kermanshah, Iran; c Research Center for Environmental Determinants of Health (RCEDH), Health Institute, Kermanshah University of Medical Sciences, Kermanshah, Iran

**Keywords:** Liquid-phase microextraction based on the solidification of deep eutectic solvent (LPME-SDES), Speciation, Blood analysis, Deep eutectic solvent, Extraction solvent lighter than water

## Abstract

In this research, a new liquid phase microextraction based on the solidification of deep eutectic solvent (LPME–SDES) has been developed for the speciation of As(III), As(V), Se(IV), Se(VI), Hg(II) and organic mercury (R-Hg) in the blood of children prior to their analysis by iridium-modified tube electrothermal atomic absorption spectrometry (ETAAS).

• In this method, a green solvent consisting of Choline chloride and decanoic acid in the molar ratio of 1:2 was used as a green hydrophobic deep eutectic solvent for the extraction of complexed ions from real blood samples.
• The DESs replace the toxic organic solvents apply for extraction and could be synthesize from cheap accessible chemicals.
• Under the optimum conditions, the calibration graphs for As, Se and Hg were linear in the rage of 0.15–40, 0.05–5.0 and 0.30–60 μg l^−1^, respectively and, the detection limit of As, Se and Hg were 0.05, 0.015 and 0.10 μg l^−1^, respectively.

## Graphical abstract

**Figure:**
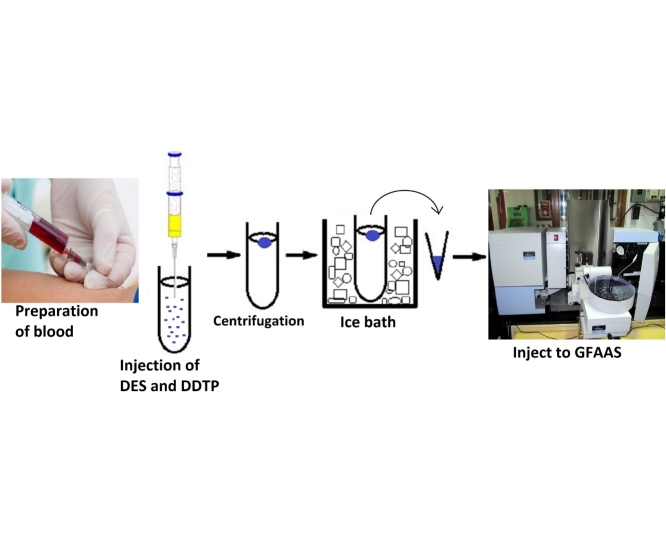

**Specification Table**

**Table:** 

Subject Area:	*Chemistry*
More specific subject area:	*Analytical Chemistry*
Method name:	*Liquid-phase microextraction based on the solidification of deep eutectic solvent (LPME-SDES)*
Name and reference of original method:	*Paiva (2014):* [3]
Resource availability:	*Not applicable*

## Method details

In recent years, a plethora of extraction techniques has emerged as environmental-friendly alternatives to conventional extraction procedures. Deep eutectic solvents (DESs), as a subclass of Ionic liquids, show comparable characteristics; they are cheaper and easy to be produced due to lower cost of the raw materials, less toxic and often biodegradable [1,2]. DESs usually provide a network of hydrogen bond acceptor (HBA) and hydrogen bond donor (HBD), thus favoring the dissolution process of target analytes [3]. DESs consist of two or three components with a melting point lower than their individual components, including HBD such as urea, glycerol, carboxylic acid, sugar, and the HBA such as quaternary ammonium salt (Choline Chloride) in a particular molar ratio [4]. DESs not only have the advantages of low volatility, low vapor pressure, high thermal stability and high ability to extract organic and inorganic compounds, but also have low cost and easy preparation of non-toxic compounds. However, DESs consist of Choline Chloride (ChCI), urea, glycerol, carboxylic acid or sugars, which have a high hydrophobicity, which affects their use in different matrices [5,6].

## Chemicals and materials

Sodium selenite and sodium selenate (both purchased from Merck, Darmstadt, Germany) were used for preparation of stock standard solutions of selenite and selenate with a concentration of 1000 mg l^−1^. Stock standard solutions of As(III) and As(V) with a concentration of 1000 mg l^−1^ were obtained by dissolving appropriate amounts of As_2_O_3_ and Na_2_HAsO_4_ (Merck, Darmstadt, Germany). An Hg (II) standard stock solution (1000 mg l^−1^ in 1% nitric acid, 250 ml) was purchased from Fluka, Buchs, Switzerland. The CH_3_Hg^+^ 1000 mg l^−1^ stock solution was prepared by dissolving appropriate amount of its chloride (Merck, Darmstadt, Germany) in the smallest possible volume of methanol and diluting to volume with deionized water. The stock standard solutions were diluted to prepared a required standard solutions with 0.1 M HCl. The analytical grade diethyldithiophosphoric acid (DDTP) as chelating agent was obtained from Merk (Darmstadt, Germany). Iridium solution with concentration of 1000 mg l^−1^ in hydrogen chloride (Sigma-Aldrich, St. Louis, Missouri, USA) was used as the chemical modifier. Decanoic acid and choline chloride were purchased from Sigma-Aldrich (St. Louis, Missouri, USA).

## Instrumentation

An Analytik Jena AG Model nov AA 400 (Jena, Germany) atomic absorption spectrometer equipped with a deuterium background correction system, a transversely heated graphite tube atomizer and a MPE60 auto-sampler were employed throughout measurements. Pyrolytic graphite coated tubes with PIN-graphite platform (Analytik Jena, Part No.:407-A81.026, Jena, Germany) were applied for the optimizations with aqueous standards. A hollow cathode lamp was used as the radiation source. Argon 99.999% was used as purge and protective gas. Integrated absorbance (peak area) was used exclusively for signal evaluation. The sample injection volume was 20 μl in all experiments. The instrumental parameters and temperature program for the graphite atomizer are listed in Table 1. The pH values were measured by Metrohm pH meter Model 692 (Herisau, Switzerland). A Multi-Wave 3000 microwave-assisted UV system (MUV, Anton Paar, Graz, Austria) was used for converting R-Hg to Hg^2+^.

**Table 1:** Instrumental analytical conditions of investigated metal ions.

Hg	Se	As	Spectrometer parameters
253.7	196.0	193.7	Wavelength (nm)
0.2	0.7	0.8	Slit width (nm)
5	8	5	Lamp current (mA)

Gas flow (ml min^−1^)	Time (s)		Temperature (°C)			Step
	Hold	Ramp	Hg	Se	As	
600	2	1	50	50	50	Pre-warming
600	15	5	80	80	80	Inject modifier
						Inject sample
600	15	10	95	90	110	Drying I
600	20	5	110	100	240	Drying II
600	10	10	210	1000	850	Ashing
0	1	0	210	1000	850	Gas stop step
0	3	0	1100	2000	2100	Atomization
1200	2	0	1500	2200	2400	Cleaning

## Sampling and sample preparation

Blood samples were collected from five children (3 girls and 2 boys) who were patient under treatment, kindly provided by the Clinic of Mohammad Kermanshahi Hospital (Kernanshah, Iran). The age was in the range 2–10 years. To preparation of sample, 1.0 ml of blood sample was placed in a 10-ml glass tube and 600 μl acetonitrile and 900 μl of 15% (w/v) ZnSO_4_ was added. The glass tube was vortexed for 5 min, maintained at 5 °C for 10 min followed by centrifugation at 5000 rpm for 3 min. Then, the supernatant was collected in another tube and this solution was diluted to 10.0 ml using ultrapure water. The resulting solution was then subjected to the LPME–SDES procedure.

## LPME-SDES procedure

An aliquot of 10.0 ml of ultra-pure water or pretreated/diluted blood sample spiked or not with target analytes was placed in a 10-ml test tube and 60 μl of DES (extraction solvent) containing 15.0 μL DDTP (chelating agent) was rapidly injected into the sample solution with a 100-μL syringe (Gastight, Hamilton, Reno, NV, USA). The mixture was then shaken using a vortex agitator for 5 min to ensure full contact of the DES and target ions inside the sample solution. The mixture was then centrifuged for 4 min at 5000 rpm in order to separate the mixture into phases. After centrifugation, the fine droplets of DES float at the top of the test tube. The test tube was then transferred into an ice bath and the DES was solidified after few minutes. Then obtained solidified DES was transferred into a conical vial where it was melted immediately. To decrease viscosity and simply injected into the GFAAS, a 20 μl acidic ethanol added into the vial. Finally, for quantitation of target ions, 30.0 μl of the extract using an auto-sampler was injected into the GFAAS and was subjected to the temperature program, shown in Table 1.

## Method validation

The analytical characteristics of the method, i.e., precision, detection limits and linearity, were investigated under the chosen experimental conditions. The results are listed in Table 2. The percent relative standard deviations (RSDs %) were between 2.2 and 4.1. The limit of detection, defined as C_L_ = 3S_B_/m (where C_L_, S_B_, and m are the limit of detection, standard deviation of the blank and slope of the calibration graph, respectively), were obtained between 0.015 and 0.10 μg l^−1^ for different metal ions. Linear ranges (LRs) of 0.15–40 μg l^−1^ for As, 0.05–5.0 μg l^−1^ for se and 0.30–60 μg l^−1^ for Hg were obtained. The correlation coefficient of the calibration curves were in the range of 0.993–0.999. The enhancement factor, obtained from the slope ratio of calibration graph after and before extraction, were in the range of 98–106.

**Table 2:** Figures of merit of the proposed method.

Analyte	Enhancement factor	Detection limit (μg l^−1^)	RSD% (intra–day, n = 7)	RSD% (inter–day, n = 7)	Linear range (μg l^−1^)	r^2^
As	106	0.05	3.2	4.7	0.15–40	0.995
Se	99	0.015	2.2	3.5	0.05–5.0	0.999
Hg	98	0.10	4.1	5.8	0.30–60	0.993

## Determination of As in blood samples

After applying the proposed methodology, total inorganic arsenic (iAs) was then measured after the reduction of As(V) to As(III) with sodium thiosulfate and potassium iodide. As(V) was calculated by the difference between the total As and As(III). As(III) and As(V) in all blood samples were detected at different concentration levels and they were confirmed by spiking As(III) and As(V) into the all samples. The concentration of As(III) and As(V) in the blood samples are shown in Table 3. Blood samples were spiked with As(III) and As(V) standards to assess matrix effects. The relative recoveries of As(III) and As(V) from blood samples at spiking different levels are listed in Table 3. In addition, the accuracy of the proposed method was evaluated by analyzing a standard reference material (SRM) NIST-2669 Inorganic Arsenic Species in Frozen Human Urine from National Institute of Standards and Technology (NIST), with certified As(III) and As(V) content of 1.47 ± 0.10 μg l^–1^ and 2.41 ± 0.30 μg l^–1^, respectively. No significant difference was found between the result obtained by employing the proposed method and the certified value (Table 3).

**Table 3:** Determination of As(III) and As(V) in blood samples, and relative recovery of spiked arsenic in these samples^a^.

samples	Analyte	Added (μg l^−1^)	Found, mean ± SD^b^ (n = 3) (μg l^−1^)	Relative recovery (%)
Blood 1 (taken from a 2-year-old girl)	As(III)	0	3.2 ± 0.16	–
		5	8.3 ± 0.41	102
		10	13.1 ± 0.8	99
	As(V)	0	2.1 ± 0.14	–
		5	7.3 ± 0.3	102
		10	11.8 ± 0.7	97

Blood 2 (taken from a 5-year-old girl)	As(III)	0	0.83 ± 0.04	–
		2	2.88 ± 0.16	103
		4	4.65 ± 0.2	95
	As(V)	0	1.20 ± 0.05	–
		2	3.08 ± 0.15	94
		4	5.23 ± 0.3	101
Blood 3 (taken from a 6-year-old girl)	As(III)	0	7.12 ± 0.4	–
		3	10.22 ± 0.6	103
		6	13.04 ± 0.5	99
	As(V)	0	4.33 ± 0.18	–
		3	7.40 ± 0.4	102
		6	10.12 ± 0.7	97

Blood 4 (taken from a 4-year-old boy)	As(III)	0	1.60 ± 0.04	–
		5	6.52 ± 0.2	98
		10	11.65 ± 0.6	100
	As(V)	0	2.4 ± 0.7	–
		5	7.3 ± 0.4	98
		10	12.2 ± 1.0	97

Blood 5 (taken from a 10-year-old boy)	As(III)	0	5.62 ± 0.2	–
		2	7.5 ± 0.4	93
		4	9.7 ± 0.3	101
	As(V)	0	1.5 ± 0.06	–
		2	3.4 ± 0.1	95
		4	5.7 ± 0.3	107

NIST SRM-2669	As(III)	1.47 ± 0.10^c^	1.43 ± 0.08	97
	As(V)	2.41 ± 0.30^c^	2.44 ± 0.22	101

a These data are based on the diluted volumes of blood samples and dilution effect was considered for calculation of them.

b Standard deviation.

c Certified values.

## Determination of Se in blood samples

Determination of total inorganic selenium is based on the conversion of Se(VI) species into Se(IV), which are then analyzed by GFAAS. Gentle boiling in 5 mol l^−1^ HCl medium for one hour and adjusting pH to 3 were used as the appropriate conditions for rapid reduction of Se(VI) to Se(IV). Finally, the concentration of Se(VI) was calculated by subtracting the Se(IV) concentration from the total inorganic selenium concentration. Results in Table 4 show that all samples contain selenite and selenate with different concentrations. To validate the method and evaluate the effects of the matrix, all samples were spiked with standard selenite and selenate solutions. Relative recovery of selenite and selenate in spiked blood samples at various concentration levels is shown in Table 4, ranging from 91 to 105%. Moreover, the accuracy of the LPME-SDES was appraised by analyzing a standard reference material (SRM) NIST-1598A inorganic constituent in frozen animal (Bovine and Porcine) serum, with certified Se content of 134.4 ± 5.8 μg l^–1^. No remarkable difference was observed between the result gained by applying the presented method and the certified value (Table 4).

**Table 4:** Determination of Se(IV) and Se(VI) in blood samples, and relative recovery of spiked Se in these samples.

Samples	Analyte	Added (μg l^−1^)	Found, mean ± SD (n = 3) (μg l^−1^)	Relative recovery (%)
Blood 1 (taken from a 2-year-old girl)	Se(IV)	0	22.5 ± 1.6	–
		15	38.1 ± 3.1	104
	Se(VI)	0	9.7 ± 0.4	–
		15	25.4 ± 2.3	105

Blood 1 (taken from a 5-year-old girl)	Se(IV)	0	21.2 ± 1.7	–
		20	39.8 ± 3.3	93
	Se(VI)	0	11.4 ± 0.6	–
		20	31.5 ± 2.4	101

Blood 1 (taken from a 6-year-old girl)	Se(IV)	0	28.0 ± 1.5	–
		23	48.5 ± 3.5	103
	Se(VI)	0	16.2 ± 0.8	–
		23	37.4 ± 2.2	92

Blood 4 (taken from a 4-year-old boy)	Se(IV)	0	40.6 ± 2.9	–
		25	63.3 ± 4.4	91
	Se(VI)	0	23.5 ± 1.6	–
		25	47.4 ± 3.2	96

Blood 4 (taken from a 10-year-old boy)	Se(IV)	0	47.3 ± 2.4	–
		30	76.6 ± 5.3	98
	Se(VI)	0	21.6 ± 1.3	–
		30	52.5 ± 4.1	103
(SRM) NIST-1598A	Total Se	134.4 ± 5.8^a^	131.3 ± 6.5	98

a Certified value.

## Determination of Hg in blood samples

Determination of total mercury (t-Hg) is based on the conversion of organic mercury(R-Hg) species into Hg^2+^, which are then analyzed by GFAAS. Ultraviolet (UV) light and microwave were used as the appropriate sources for rapid conversion of R-Hg to Hg^2+^. In this work, we used MUV 3000, a closed vessel microwave assisted-UV system, equipped with both the thermal and radiant (UV) energies. Total Hg was obtained when the aqueous samples containing both Hg^2+^ and R-Hg was put on vessel in special conditions (180 °C, UV) for 15 min. All R-Hg species were converted to Hg^2+^. Finally, the concentration of R-Hg was calculated by subtracting the Hg^2+^ concentration from the t-Hg concentration. Mercury in all blood samples were detected at different concentration levels (Table 5). Blood samples were spiked with Hg^2+^ standard solution to assess matrix effects. The results of relative recovery and the concentrations obtained in spiked studies of the blood samples are also included in Table 5. In addition, the accuracy of the proposed methodology was evaluated by analyzing a standard reference material (SRM) NIST-955C Toxic Metals in Frozen Caprine Blood, with certified contents of 9.0 ± 1.3 and 17.8 ± 1.6 μg l^–1^ inorganic mercury and total mercury, respectively. No significant difference was found between the results obtained by employing the proposed method and the certified value. The determined value of 8.6 ± 1.4 μg l^–1^ for inorganic mercury and 18.2 ± 1.5 μg l^–1^ for total mercury are in satisfactory agreement with the certified values.

**Table 5:** Determination of Hg^2+^, R-Hg and t-Hg in blood samples and relative recovery of spiked mercury in these samples.

samples	t-Hg (μg l^−1^)	Analyte speciation	Added (μg l^−1^)	Found, mean ± SD (n = 3) (μg l^−1^)	Relative recovery (%)	
Blood 1 (taken from a 2-year-old girl)	2.02	Hg^2+^	0	0.82 ± 0.13	–	
			2	2.90 ± 0.21	104	
			3	3.61 ± 0.25	93	
		R-Hg	0	1.20 ± 0.05	–	
			2	3.31 ± 0.26	105	
			3	4.10 ± 0.22	97	

Blood 1 (taken from a 5-year-old girl)	1.40	Hg^2+^	0	0.44 ± 0.05	–	
			3	3.23 ± 0.17	93	
			4	4.51 ± 0.32	102	
		R-Hg	0	0.96 ± 0.07	–	
			3	4.15 ± 0.25	106	
			4	4.74 ± 0.26	94	

Blood 1 (taken from a 6-year-old girl)	3.35	Hg^2+^	0	2.03 ± 0.15	–	
			1	3.12 ± 0.20	109	
			2	3.90 ± 0.30	93	
		R-Hg	0	1.32 ± 0.10	–	
			1	2.40 ± 0.16	108	
			2	3.22 ± 0.22	95	

Blood 1 (taken from a 4-year-old boy)	2.06	Hg^2+^	0	0.56 ± 0.04	–	
			2	2.35 ± 0.10	90	
			3	3.63 ± 0.15	102	
		R-Hg	0	1.50 ± 0.12	–	
			2	3.30 ± 0.40	90	
			3	4.71 ± 0.25	107	

Blood 1 (taken from a 10-year-old boy)	3.65	Hg^2+^	0	1.10 ± 0.13	–	
			4	5.00 ± 0.28	97	
			5	6.22 ± 0.33	102	
		R-Hg	0	2.55 ± 0.24	–	
			4	6.39 ± 0.50	96	
			5	7.61 ± 0.46	101	

## References

[1] Shishov A., Bulatov A., Locatelli M., Carradori S., Andruch V. (2017). Application of deep eutectic solvents in analytical chemistry. A review. Microchem. J..

[2] Cunha S.C., Fernandes J.O. (2018). Extraction techniques with deep eutectic solvents. Trends Anal. Chem..

[3] Paiva A., Craveiro R., Aroso I., Martins M., Reis R.L., Duarte A.R.C. (2014). Natural deep eutectic solvents–solvents for the 21st century. ACS Sustain. Chem. Eng..

[4] Akramipour R., Golpayegani M.R., Gheini S., Fattahi N. (2018). Speciation of organic/inorganic mercury and total mercury in blood samples using vortex assisted dispersive liquid-liquid microextraction based on the freezing of deep eutectic solvent followed by GFAAS. Talanta.

[5] Akramipour R., Golpayegani M.R., Ghasemi M., Noori N., Fattahi N. (2019). Development of an efficient sample preparation method for the speciation of Se(IV)/Se(VI) and total inorganic selenium in blood of children with acute leukemia. New J. Chem..

[6] Faraji M. (2019). Determination of some red dyes in food samples using a hydrophobic deep eutectic solvent-based vortex assisted dispersive liquid-liquid microextraction coupled with high performance liquid chromatography. J. Chromatogr. A.

